# Restoration Skills Training in a Natural Setting Compared to Conventional Mindfulness Training: Sustained Advantages at a 6-Month Follow-Up

**DOI:** 10.3389/fpsyg.2022.763650

**Published:** 2022-08-01

**Authors:** Freddie Lymeus, Mathew P. White, Per Lindberg, Terry Hartig

**Affiliations:** ^1^Institute for Housing and Urban Research, Uppsala University, Uppsala, Sweden; ^2^Department of Psychology, Uppsala University, Uppsala, Sweden; ^3^Vienna Cognitive Science Hub, University of Vienna, Vienna, Austria

**Keywords:** mindfulness, restoration, nature, meditation, health, follow-up, acceptability, compliance

## Abstract

Restoration skills training (ReST) is a mindfulness-based course in which participants draw support from a natural practice setting while they learn to meditate. Well-established conventional mindfulness training (CMT) can improve psychological functioning but many perceive it as demanding and fail to sustain practice habits. Applying non-inferiority logic, previous research indicated that ReST overcomes compliance problems without compromising the benefits gained over 5 weeks’ training. This article applies similar logic in a 6-month follow-up. Of 97 contacted ReST and CMT course completers, 68 responded and 29 were included with multiple imputation data. The online survey included questions about their psychological functioning in three domains (dispositional mindfulness, cognitive lapses, and perceived stress) and the forms and frequencies with which they had continued to practice mindfulness after the course. Former ReST participants continued, on average, to show higher dispositional mindfulness and fewer cognitive lapses compared to pre-course ratings. Improved psychological functioning in one or more domains was demonstrated by 35%, as determined by a reliable change index. Again, analyses detected no indications of any substantive disadvantages compared to the more demanding, established CMT approach. Compared to the CMT group, more ReST participants had also continued to practice at least occasionally (92 vs. 67%). Continued practice was linked to sustained improvements for ReST but not clearly so for CMT. ReST participants thus continued to use the skills and sustained the improvements in psychological functioning that they had gained in the course, further supporting the utility of ReST as a health intervention.

## Introduction

Restoration skills training (ReST) is a mindfulness-based meditation training course in which the practice approach draws support from a setting rich in natural features (Lymeus, 2019). In our studies, university students with stress or concentration problems participated in ReST courses in an urban botanical garden. This article presents a 6-month follow-up with the same measures of psychological functioning used to assess outcomes directly after the course (see Lymeus et al., 2020). Before presenting the study, we briefly review the arguments for ReST and previous findings that motivate the long-term follow-up.

### Arguments for ReST

The various garden settings where we gave the ReST courses have high restorative quality (Dahlöf and Toll, 2020), as many natural settings do. Specifically, natural settings often provide a sense of being away from stressors and mental routines, and contain softly fascinating features that hold attention effortlessly (Kaplan, 1995, 2001). These qualities presumably mediate restoration of weakened cognitive self-regulation capabilities (i.e., the ability to willfully direct attention; see Ohly et al., 2016; Stevenson et al., 2018), promotion of positive emotions (see McMahan and Estes, 2015), and reduction of stress (see Bowler et al., 2010). These processes and outcomes resemble processes and outcomes in mindfulness, which involves attentiveness to present experience with qualities of decentering (i.e., psychological detachment) and curiosity (i.e., openness and acceptance; Bishop et al., 2004; also see Lymeus, 2019; Macaulay et al., 2022), and is known to improve attention regulation and reduce stress (Sedlmeier et al., 2018; Cásedas et al., 2019).

However, well-established conventional mindfulness training (CMT) approaches for beginners [e.g., Mindfulness-Based Stress Reduction (MBSR); Kabat-Zinn, 1990] are typically conducted in sparsely furnished indoor settings. There, they use focusing exercises that target internal aspects of experience, with the aim to train the involved skills and brain networks rather than restore access to existing self-regulation capabilities (Lutz et al., 2008; Tang et al., 2015; Fox et al., 2016). In such training, progress presumably relies on regular practice with given meditation exercises several times per week. Struggling to maintain focus and other transient experiences of cognitive effort during exercises have been described as harmless and possibly necessary parts of the process of learning to regulate attention more efficiently (Malinowski, 2013; Lutz et al., 2015; Baer et al., 2019). Many beginners also struggle to find time and space for uninterrupted meditation (Birtwell et al., 2018). In theory and practice, then, the conventional approach to mindfulness does not involve notions of how environments can support regulation and restoration of attention but rather assumes that regular, short-term investments of effort in exercises will improve base-levels of available resources. Once acquired, the enhanced skills and brain functions can be useful in daily life situations where they help a person regulate behavior and prevent and manage stressors more efficiently. Accordingly, CMT is known to yield benefits in multiple domains of psychological functioning, including attention regulation and chronic stress (Sedlmeier et al., 2018; Cásedas et al., 2019).

In contrast, the practice approach in ReST uses adapted mindfulness instructions that help participants connect with sensory impressions from the environment and draw on supporting restorative processes. While the ReST classes in this study were held in a botanical garden, we assume that many settings with natural features of some restorative quality could support such mindful states and practices. Most people in Europe and many other parts of the world can access natural environments on a regular basis (Van den Berg et al., 2007; Korpela et al., 2018; White et al., 2021). The expectation is that training with ReST can teach people how to use such settings to manage and restore attention resources and regulate stress in a more efficient yet effortless way (*cf.* Kaplan, 2001; Tang and Posner, 2009; Macaulay et al., 2022). For instance, we expect that participants who complete the course are more capable of noticing needs for restoration, of seeking out settings that support restoration, and of engaging with those settings in ways that support restorative processes. Given that ReST draws on restoration processes, we also expect that the training will be less demanding and more appealing than with a conventional approach to mindfulness training, so larger numbers will establish and sustain mindfulness habits. Even without relying on effortful training, however, we expect participants to learn widely applicable mindfulness skills and use these preemptively to support health and prevent unnecessary stress. Previous research comparing ReST to formally matched CMT conducted indoors has largely affirmed these expectations in connection with the 5-week courses.

### Previous Comparisons of ReST and Conventional Mindfulness Training

Lymeus et al. (2018) analyzed attentional performance tests obtained directly before and after meditation practice on weeks 1, 3, and 5 of ReST and CMT courses. ReST participants consistently improved in general attention performance from before to after practice, consistent with low effort and the environmental support for restoration (Kaplan, 1995; Hartig, 2007). In contrast, CMT participants increasingly deteriorated in performance across the weeks, consistent with assumptions that conventional mindfulness exercises incur effort as beginners try to learn and apply the relevant skills. Regarding executive attention, ReST participants increasingly improved with the meditation, indicating that the training gradually taught them to enhance restorative processes in relation to the environment. In contrast, CMT participants’ executive performance was unaffected by the training. These findings suggest that ReST, which uses exercises meant to minimize effort and support restoration, is a less demanding introduction to mindfulness training than CMT. This matters because mindfulness training is frequently recommended to people with stress and concentration problems as a means to enhance their psychological functioning (Brown et al., 2007; Holzel et al., 2011; Baer et al., 2012); however, compliance with the practice recommendations is often problematically low in conventional mindfulness interventions (Nam and Toneatto, 2016), particularly among participants with more pronounced attention regulation problems (Crane and Williams, 2010; Lymeus et al., 2017). Those participants plausibly have elevated sensitivity to undesired effort with the training (*cf.* Sekhon et al., 2017; Baer et al., 2019).

In further work, Lymeus et al. (2019) proceeded to investigate compliance differences and showed that ReST participants had lower drop-out during the course and practiced more consistently with the assigned meditation homework than CMT participants. Compliance differences were serially mediated through experiences of restorative environmental qualities and state mindfulness during the classes. Apparently, ReST participants felt more detached from everyday pressures and routines and more positively engaged with present experiences, presumably making continuation more rewarding and less effortful than with CMT.

Yet, if regular effortful training drives improvements in psychological functioning with CMT, as established mindfulness theory suggests (Lutz et al., 2008; Holzel et al., 2011; Malinowski, 2013), the less demanding ReST could confer less benefit due to lower skill development. Addressing such concerns, Lymeus et al. (2020) evaluated change from before to after ReST and CMT courses in ratings of three domains of psychological functioning: dispositional mindfulness (Five Facet Mindfulness Questionnaire; Baer et al., 2006), the occurrence of cognitive mistakes or lapses (Cognitive Failures Questionnaire; Broadbent et al., 1982), and perceived stress (Perceived Stress Scale; Cohen et al., 1983), all of which were also assessed in the follow-up reported here. On average, both ReST and CMT participants improved similarly over the 5 weeks, with more consistent improvements in dispositional mindfulness and reductions in cognitive lapses than reductions in perceived stress.

Lymeus et al. (2020) also went further to assess whether individual-level change in the measures reflected meaningful and reliable change in functioning beyond possible measurement error by comparing ReST and CMT using a reliable change index. The reliable change index is a common metric in clinical trials that seek to evaluate the practical utility of a treatment (see Jacobson and Truax, 1991; Wise, 2004). It specifies how much change in a score must be observed for 95% confidence that a given individual has undergone actual change in the measured construct, given the reliability of the particular measure. Analyses using a reliable change index complement analyses of group average scores because the group average can be significantly improved although only few participants experienced any substantive improvement, and even when many participants actually deteriorated. These reliable change analyses showed that over one third of the individual ReST and CMT participants achieved reliably improved psychological functioning in one or more domains. The proportion that showed deteriorated functioning was small and similar to that observed in a passive control group (a pattern that corresponds well with other mindfulness studies that have reported on undesired outcomes; see Baer et al., 2019; Aizik-Reebs et al., 2021).

Lymeus et al. (2020) also applied the logic of non-inferiority tests, another approach common in clinical trials (see Snapinn, 2000; Schumi and Wittes, 2011). Non-inferiority testing is the appropriate way of evaluating the utility of a treatment that has been modified, for instance in terms of a more efficient or acceptable delivery format (i.e., ReST), compared to an established alternative (i.e., CMT) to ensure that the advantages of the modifications outweigh any potential disadvantages (i.e., poorer outcomes). Evaluations of non-inferiority thus differ from evaluations of superiority (as normally conducted with tests for statistically significant differences), from null findings (as in the absence of statistically significant differences), and from the rarely warranted conclusion that there is no difference at all. Instead, non-inferiority tests compare observed group differences against a criterion regarding how much possible disadvantage in terms of outcomes is acceptable in light of the specific advantages of the new treatment compared to the established one. Criteria are based on the statistical magnitude or practical meaning of a given effect, rather than on significance tests. To establish a useful criterion, Lymeus et al. (2020) built on the findings observed by Lymeus et al. (2019) that ReST had moderate-sized advantages over CMT in that more participants completed it (*φ* = 0.211) and maintained steady meditation habits during the course (*η_p_^2^* = 0.046). They also considered commonly used statistical benchmarks for the “minimal important difference” in self-reported health outcomes (i.e., the smallest effect that is attended by perception of a meaningful change, where cutoffs typically fall between 0.2 and 0.5 SD; see, e.g., Revicki et al., 2008; Mouelhi et al., 2020). Hence, they settled on a quite conservative criterion of 0.2 SD (i.e., *ƞ_p_^2^* = 0.01 in ANOVA, *φ* = 0.10 in 1 df Chi-square tests; equivalent to a small effect according to the tentative guidelines provided by Cohen (1988a) and argued that if ReST produced outcomes that fell short of those of CMT by no more than that, then ReST retained a practically meaningful part of its known advantage over CMT in terms of compliance, by allowing larger numbers to enjoy the benefits of the training. Furthermore, such a minimal disadvantage would most likely be inconsequential to participants. Using this criterion, Lymeus et al. (2020) found no indication that ReST produced meaningfully poorer outcomes than CMT. In sum, the lower effort needed for ReST was associated with several advantages compared to CMT (as described in Lymeus et al., 2018, 2019), but with no apparent disadvantages.

### The Present Study

This study further assesses the utility of ReST compared to CMT by analyzing data obtained 6 months after course completion. It is essential to study the degree to which practice habits and benefits achieved with ReST are sustained over time as well as any potential long-term drawbacks of using this novel integration of environmental and mindfulness-based approaches, in order to determine its suitability and utility as a health intervention. More broadly, longitudinal follow-ups of this kind are important because personal and societal investments in health interventions are motivated by prospects of sustainable gains; yet, few studies of mindfulness interventions have followed participants’ long-term commitment, continued development, and possible negative experiences after completing an introductory course (e.g., Davidson and Kaszniak, 2015; Van Dam et al., 2018; Baer et al., 2019). In research on nature-based health interventions, generally, the understanding of capacity-building mechanisms beyond short-term effects of restoration or other transient processes is underdeveloped (Dzhambov et al., 2019).

For nature-based interventions to gain acceptance in health promotion and treatment, there is a need for studies that compare them to established approaches with known benefits rather than to passive or sham conditions, and with methods suited for evaluating their practical utility (e.g., Annerstedt and Währborg, 2011; Djernis et al., 2019). Such methods include evaluation of both positive and negative change on the individual level (e.g., using a reliable change index) in addition to commonly studied average effects. Furthermore, comparisons against established approaches must consider that the expected advantages of nature-based interventions may often inhere to processes (e.g., restoration, acceptability, and compliance) rather than to markedly enhanced efficacy in promoting distal health outcomes. For instance, while physical exercise completed in nature may be similarly effective as indoor exercise in promoting physical health, the fact that some groups are more willing to exercise outdoors means greater overall advantages (Elliott et al., 2015; White et al., 2016; Lahart et al., 2019). However, similarity cannot be assessed with conventional statistical tests and must instead be evaluated with non-inferiority logic, based on a criterion for what level of disadvantage for either condition should be considered inconsequential or acceptable.

We used the reliable change index and the non-inferiority logic that were previously applied by Lymeus et al. (2020) to evaluate how well change was sustained 6 months after completion of the ReST and CMT courses. These follow-up analyses build on assumptions that both ReST and CMT were attended by improved psychological functioning and seek to determine whether the less demanding ReST course had meaningfully poorer outcomes than the conventional course. Following the same arguments as Lymeus et al. (2020, detailed above in the section “Previous Comparisons of ReST and Conventional Mindfulness Training”), we evaluated non-inferiority against the criterion of *ƞ_p_^2^* < 0.01 or *φ* < 0.10 (commonly considered a small effect) to the disadvantage of ReST compared with CMT in the measures of psychological functioning: If the sustained benefits 6 months after completing ReST fall within this small margin, ReST retains a meaningful advantage overall given that larger numbers went through the course successfully. Furthermore, participants would likely perceive the two courses as equally effective.

We also followed-up on continued practice in the 6 months since the course. Building on the previous findings by Lymeus et al. (2019) that ReST participants dropped out less and practiced more consistently during the course itself, we expected ReST to still be superior (rather than merely non-inferior) to CMT in this regard, and applied conventional statistical significance criteria.

We tested four hypotheses:

*H1*: We expected improvements in psychological functioning compared to before the course for the group average scores of both ReST and CMT, in terms of greater dispositional mindfulness (H1a), fewer cognitive lapses (H1b), and reduced perceived stress (H1c).
*H2*: We expected that ReST would not be inferior to CMT in terms of average improvement. Specifically, the effect size of the average group difference in improvement would be no more than *ƞ_p_^2^* = 0.01 to the disadvantage of ReST compared with CMT, regarding dispositional mindfulness (H2a), cognitive lapses (H2b), and perceived stress (H2c).
*H3*: We expected that ReST would not be inferior to CMT in terms of the proportions of participants that showed reliably improved or deteriorated psychological functioning compared to before the course, as determined by the reliable change index. Specifically, the effect size of the group difference in proportions classified as reliably changed would be no more than *φ* = 0.10 to the disadvantage of ReST compared with CMT, regarding improvement (H3a) and deterioration (H3b).
*H4*: We expected that the proportion of ReST participants who continued to practice mindfulness after the course would be higher than that of CMT participants. We tested this separately for two operationalizations of continued practice: occasional practice (having practiced at least several times vs. discontinuing the practice; H4a), and regular practice (having practiced at least once per week; H4b).

We also sought to illuminate the role that continued practice played in sustaining improvements in psychological functioning achieved with the course over the 6-month follow-up period. Specifically, we considered the relationships between improvements observed directly after the course, continued mindfulness practice in the following 6 months, and improvements observed at follow-up. The relevance is to compare the degree to which improvements observed at follow-up were sustained from the course and explained by continued mindfulness practice rather than factors external to the study. The rationale thus connects to mindfulness theory indicating that practice is an important driver of benefits as well as to previous findings that ReST participants practiced more consistently than CMT participants during the course itself. However, we did not have any theoretically derived expectations that ReST and CMT would differ in specific ways with regard to the role of continued practice.

## Materials and Methods

### Participants and Procedures

**Figure 1:**
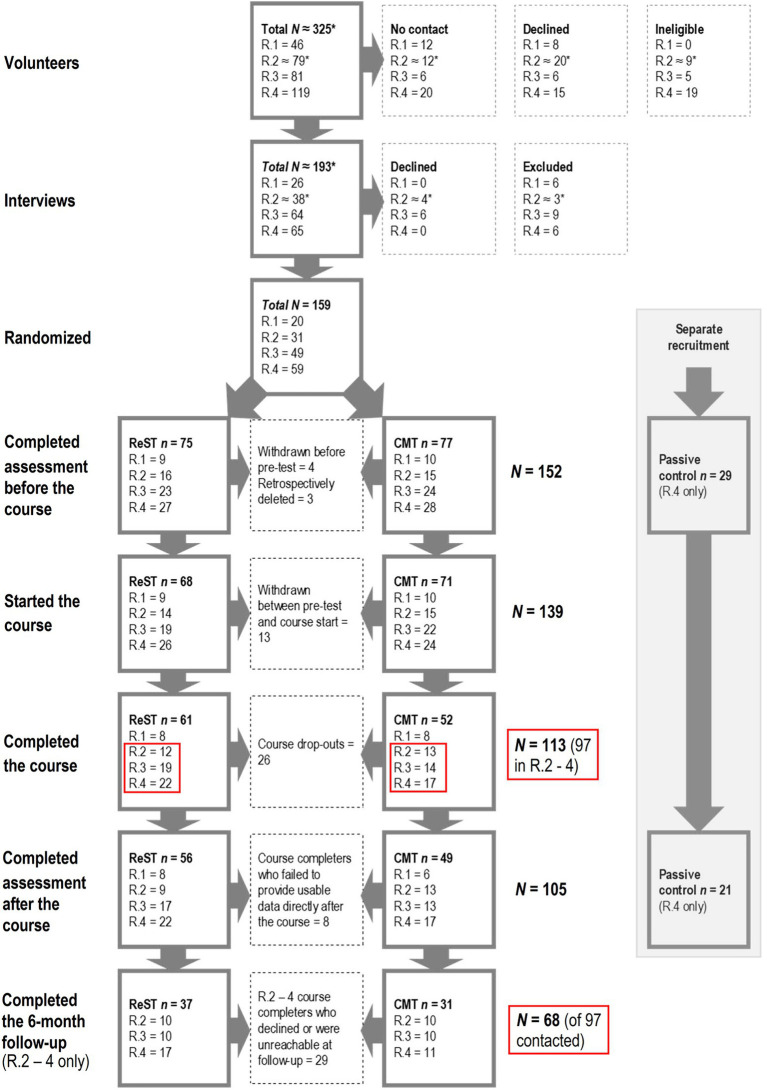
Participant flow through the recruitment, interventions, and evaluations in the four data collection rounds of the study, where participants in rounds 2–4 were contacted for the 6-month follow-up. * denotes approximations necessitated by incompleteness of the records from early stages of the recruitment for data collection round 2.

The study was approved by the Regional ethical review board for Uppsala (diary number: 2013/033) and complied with the Declaration of Helsinki. Participant flow through the enrollment, courses, assessments after the course, and assessments in the follow-up is summarized in Figure 1. Across four different rounds of data collection, we recruited 159 university students with stress or concentration problems for a study about mindfulness training (see Lymeus et al., 2020). They were informed that the study involved participation in a 5-week mindfulness course and different assessments in connection with the course, and that they could be contacted for a follow-up after 6 months. We randomly assigned them to ReST or CMT without disclosing that the study involved a contrast between training conducted in different environments. We also asked them not to discuss the course or study with anyone outside their own course group. Seventeen withdrew before the course start and three later requested their data be removed from our files. We thus have baseline data from 139 people who started the course.

Participants completed ratings of dispositional mindfulness, cognitive lapses, and perceived stress online shortly before the course started. They then received training in five 90-min classes spread over 5 weeks. The classes were given in small groups of ≤12 participants. Participants also received homework assignments to complete specified meditation exercises on most days between the classes. At the end of the course, both ReST and CMT participants were encouraged in general terms to continue practicing according to the same principles that were taught in the course. However, they received no particular recommendation or instruction to uphold a specific form or frequency of practice, nor were they specifically informed that we would follow up on continued practice.

After 5 weeks, 113 participants had completed the course (ReST = 61, CMT = 52). For the follow-up, we only contacted course-completers from data collection rounds 2–4 (*N* = 97, see Lymeus et al., 2020) because resources for a follow-up were not yet in place at the time of round 1. Participants were contacted by the e-mail address they had provided during recruitment and asked to give an update on their progress through a short online survey. They were offered a cinema ticket as thanks for continued participation. Twenty-nine declined or failed to respond, leaving 68 who provided follow-up data: ReST = 37 (26 females, median age = 24), CMT = 31 (21 females, median age = 24). To complement analyses of the observed data, we used multiple imputation to generate 30 datasets comprising all 97 eligible participants: ReST = 53 (32 females, median age = 24), CMT = 44 (30 females, median age = 24).

We checked the baseline balance in the measures of psychological functioning between those who responded to the follow-up and those who did not, and found no indications of systematic differences whether considering all 139 course starters (see Supplementary Tables S1, S2) or only those 97 who eventually were contacted for the follow-up (see Supplementary Tables S3, S4).

### Courses and Settings

#### Restoration Skills Training

Restoration skills training participants attended classes in the Uppsala botanical garden: an urban botanical garden adjacent to several university campus buildings. In data collection rounds 2–4 (which were included in the follow-up), all guided exercise, theoretical discussions and homework consultation were conducted in a tropical greenhouse. We used three larger rooms featuring dispersed canopy trees and flowering plants, winding gravel paths through dense rainforest vegetation, and a large circular water lily pond, respectively. The climate varied with a temperature range of *circa* 16°C–27°C and relative humidity of *circa* 60–90%. Small fish inhabited the several water bodies and tropical frogs hid in the vegetation. The chirping frogs, water drops hitting different surfaces, mechanical sounds from the climate system, and outside sounds emanating from weather and traffic characterized the auditory environment. In pilot studies, these settings received high ratings of restorative quality (as summarized and further supported by Dahlöf and Toll, 2020).

The training involved exercises and a rationale that directed participants to interact with the environment in ways thought to support mindfulness as well as restoration. In all its elements, ReST drew on environmental support for attentiveness to present experience, decentering, and curiosity, as well as for theoretical understanding of mindfulness principles. The basic approach involved brief grounding in bodily sensations and the breath followed by exploration of different sensory impressions in the environment. Some exercises involved walking meditation and elements of unguided exploration in the environment. For further details, see Lymeus (2019).

#### Conventional Mindfulness Training

Conventional mindfulness training participants attended classes in an adjacent campus building, in a classroom that, building on pilot studies, was expected to neither interfere with nor particularly facilitate their training (see Dahlöf and Toll, 2020). We cleared the center of the rooms from desks and arranged a circle of chairs for seating during the classes and exercises. The rooms had closed curtains and no decorations. Faint sounds could be heard, emanating from other activities in the building and traffic. The training built on MBSR (Kabat-Zinn, 1990) and emphasized inwardly focused exercises targeting bodily sensations, thoughts, and emotions. Some exercises involved standing and walking meditation practices, which were completed in the same room. For further details, see Lymeus (2019).

### Measures

For psychological functioning, we adopted the three self-report measures that were used directly before and after the course, and treated them in the same way as Lymeus et al. (2020). For each scale, we used the simple deltas of scale means for analyses of average effects. We also calculated the reliable change index (RCI; Jacobson and Truax, 1991; Wise, 2004) for each scale using the corrected formula provided by Christensen and Mendoza (1986). The RCI was derived from the test–retest reliability that Lymeus et al. (2020) observed in a separately recruited passive control group that was included in data collection round 4 of the study (but not included in the follow-up) and the observed standard deviations of the course participants’ initial scores on the respective measures. The reliable change index specifies how much change in scores must be observed for 95% confidence that an individual participant has undergone actual change in the measured construct, beyond variation that could be due to measurement error. Furthermore, we obtained measures of continued practice after the course.

#### Dispositional Mindfulness

We measured dispositional mindfulness with the Five Facet Mindfulness Questionnaire (FFMQ; Baer et al., 2006; Sauer et al., 2013). The version of FFMQ (Lilja et al., 2011) had 29 statements about how often in the last month a person had different mindfulness experiences (1 = never, 5 = always) with high internal consistency before the course (Omega total = 0.97, Cronbach’s *α* = 0.85) and at follow-up (Omega total = 0.97, *α* = 0.88). Higher scores indicate higher dispositional mindfulness. The RCI was determined to 0.65 points change in the mean item rating on the five-point scale.

#### Cognitive Lapses

We measured cognitive lapses with the Cognitive Failures Questionnaire (CFQ; Broadbent et al., 1982; Carrigan and Barkus, 2016). The CFQ has 25 questions about how often in the last month a person made different mistakes in perception, action, and memory (0 = never, 4 = very often) with high internal consistency before the course (Omega total = 0.96, *α* = 0.87) and at follow-up (Omega total = 0.95, *α* = 0.91). Higher scores indicate higher occurrence of cognitive lapses and thus poorer cognitive functioning. The RCI was determined to 0.54 points change in the mean item rating on the five-point scale.

#### Perceived Stress

We measured perceived stress with the Perceived Stress Scale (PSS; Cohen et al., 1983; Cohen, 1988b). The PSS has 14 questions about how often in the last month a person had different experiences of overload, loss of control, and inability to cope (0 = never, 4 = very often) with high internal consistency before the course (Omega total = 0.92, *α* = 0.83) and at follow-up (Omega total = 0.89, *α* = 0.88). Higher scores indicate higher perceived stress. The RCI was determined to 0.72 points change in the mean item rating on the five-point scale.

#### Continued Practice

We measured continued practice in the 6 months following the course with three questions: how often since the course ended participants had completed formal meditation, completed informal meditation, and used mindfulness in daily life. Responses were made with an ordinal scale (*Never*, *Just the odd occasion*, *Several times*, *At least once per month*, *At least once per week*, *On most days*, and *Practically every day*). These responses were not normally distributed. We dichotomized them in two distinct ways, representing two different operationalizations of continued practice. We considered it relevant to compare how many ReST and CMT participants had continued to use what they had learned at least occasionally so as to feasibly be able to handle particular situations more efficiently. We therefore classified the participants according to whether they had continued to practice *Several times* or more (1) vs. those who answered *Just the odd occasion* or *Never* (0) and thus had practically discontinued mindfulness practice after the course. We also considered it relevant to compare how many ReST and CMT participants had upheld a regular mindfulness practice (as per the recommendations and assumptions in common mindfulness courses such as MBSR) and thus classified each participant according to whether they had practiced *At least once per week* or more (1) vs. those who practiced less (0).

### Design and Statistical Analyses

Outcomes were: “change from before the course to follow-up” (continuous: delta of means; categorical: reliable change classification) and “continued practice” [categorical: occasional (yes/no), regular (yes/no)]. The study had two between-subjects factors: Course type (ReST, CMT) and data collection round (2–4). However, preliminary analyses indicated that data collection round did not impact results substantively so it was not included in final models presented here. We only analyzed data per-protocol (i.e., for course completers) because we could not obtain follow-up data from course drop-outs for intention-to-treat analyses. Analyses of the observed data regarding psychological functioning (obtained from 70% of the contacted sample) were complemented by analyses using multiple imputation of the missing values. Thirty imputed datasets were generated, where missing values were predicted independently for the two course types using these predictor variables: gender, age, initial values for the measures of psychological functioning obtained before the course, change observed directly after the course, and the intercepts and linear regression slopes for homework practice completed during the course weeks. The multiple imputations were done in RStudio using the “mice” package (v. 3.13.0). Analyses of both the observed and the imputed datasets were performed in SPSS (v. 26). For the imputed datasets, the obtained test statistics were pooled in RStudio using the “miceadds” package (v. 3.11-6). In the Results section, we provide the pooled estimates of descriptive and test statistics along with the data from analyses of the observed sample for comparison.

The data underlying the analyses will be made available following a 2-year embargo period, through the Swedish National Data Service.^1^ We analyzed the data in four steps.

#### H1 and H2: Average and Non-inferior Improvement

We used ANCOVA’s with the change scores (delta of means) for FFMQ, CFQ, and PSS as outcomes, Course type as a between-subjects factor, and the initial score obtained before the course as a covariate. Using change scores in ANCOVA to establish a difference from baseline, or a group difference in difference from baseline, is equivalent to using follow-up scores as the outcome (although the effect of the covariate will generally be larger; see Vickers and Altman, 2001; Clifton and Clifton, 2019). However, it eases interpretation because change can be evaluated as mean increase or decrease in scores and CIs can be compared against zero rather than baseline values.

We tested H1 by checking whether the 95% CIs for the estimated marginal means of the change scores overlapped with zero, and H2 by scrutinizing the effect sizes for the Course type difference. Following arguments put forth in the introduction, we considered that any disadvantage for ReST compared with CMT could be no more than *ƞ_p_^2^* = 0.01 to support conclusions regarding non-inferiority, given that ReST should retain some overall advantage based on its moderately higher compliance rates.

#### H3: Proportions of Participants Showing Reliable Improvement or Deterioration

Participants with change scores larger (positive or negative) than the determined RCI for a given measure were classified as improved or deteriorated and those with smaller change scores were classified as unchanged. For an overall index building on all three measures, we classified each participant as either improved (i.e., improved on one or more of the measures and not deteriorated on any other measure [1]), unchanged (unchanged on all measures [0]), or deteriorated (deteriorated on one or more of the measures [−1]).

We used 2 × 2 Chi-Square analysis to compare the distributions for ReST and CMT on the overall reliable change classification building on all three measures; that is, how many participants were improved [1] vs. unchanged or deteriorated [0], or deteriorated [−1] vs. unchanged or improved [0] in psychological functioning compared to before the course. To establish non-inferiority in keeping with H3, we considered that any disadvantage for ReST compared with CMT could be no more than *φ* = 0.10 for the proportions classified as improved or deteriorated, respectively.

#### H4: Proportions of Participants Who Continued to Practice Mindfulness

We used 2 × 2 Chi-Square analyses to compare the proportions of ReST and CMT participants who continued to practice mindfulness in some form after the course: one for occasional practice and one for regular practice. Superiority of ReST over CMT, per H4, was determined by the conventional criterion (*p* < 0.05). We also conducted supplementary analyses to reveal in what form(s) of continued practice ReST and CMT participants differed: formal, informal, and/or use in daily life. These were intended to provide additional insights rather than test specific hypotheses.

#### Explaining Sustained Improvement

We used 2 × 2 × 2 Chi-square analyses to determine the relationships between improvements observed directly after the course, continued mindfulness practice in the intervening 6 months, and improvements observed at the 6 month follow-up among ReST and CMT participants. Improvement at 6-month follow-up built on the reliable change classifications described above and thus distinguished participants who showed improvements in psychological functioning compared to before the course (versus unchanged or deteriorated functioning). Improvement directly after the course (versus unchanged or deteriorated functioning) built on data reported by Lymeus et al. (2020) who calculated and handled the reliable change index the same way as in this study. Continued practice was also entered as a dichotomous variable (yes = 1, no = 0). Where relevant, we repeated the analyses for continued at least occasional practice and continued regular practice.

## Results

### H1 and H2: Average and Non-inferior Improvement in Psychological Functioning

**Figure 2:**
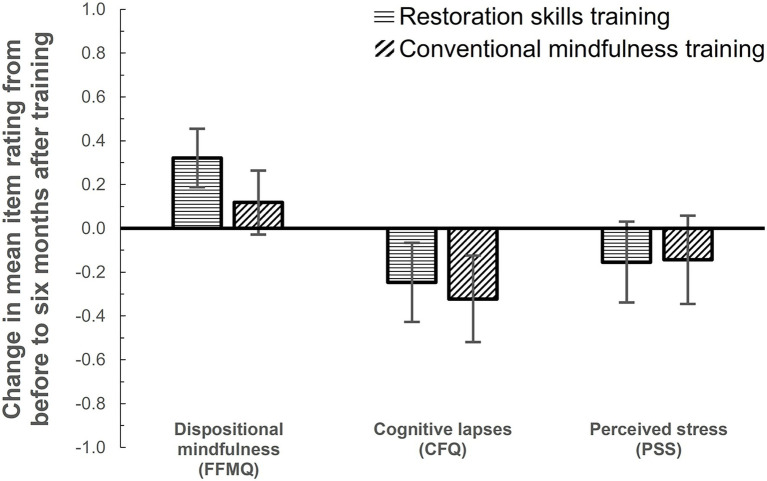
Average levels (estimated marginal means) and 95% CIs of change observed in the 6-month follow-up compared to the initial assessments before the course started, for mean item ratings of dispositional mindfulness (Five Facet Mindfulness Questionnaire; possible range 1–5), cognitive lapses (Cognitive Failures Questionnaire; possible range 0–4), and perceived stress (Perceived Stress Scale; possible range 0–4). Pretest scores obtained before the course on the respective measures were entered as covariates. The figure reflects the observed data obtained from 37 ReST participants and 31 CMT participants (estimates based on the multiple imputation datasets with *N* = 97 were very similar).

Supporting H1a and H1b, CIs showed that 6 months after course completion, ReST participants still demonstrated, on average, sustained improvements in dispositional mindfulness (observed *M* = 0.32, 95% CI [0.19, 0.46]; imputed *M*_pooled_ = 0.29, 95% CI [0.18, 0.41]) and fewer cognitive lapses (*M* = −0.25, 95% CI [−0.43, −0.07]; imputed *M*_pooled_ = −0.21, 95% CI [−0.39, −0.04]; see Figure 2). Contrary to H1c, however, there was no sustained reduction in perceived stress (*M* = −0.15, 95% CI [−0.34, 0.03]; imputed *M*_pooled_ = −0.14, 95% CI [−0.31, 0.03]). CMT participants demonstrated, on average, fewer cognitive lapses (*M* = −0.32, 95% CI [−0.52, −0.12]; imputed *M*_pooled_ = −0.29, 95% CI [−0.48, −0.11]) but not improved dispositional mindfulness (*M* = 0.12, 95% CI [−0.03, 0.26]; imputed *M*_pooled_ = 0.11, 95% CI [−0.03, 0.25]) or reduced perceived stress (*M* = −0.14, 95% CI [−0.35, 0.06]); imputed *M*_pooled_ = −0.14, 95% CI [−0.35, 0.07]. Table 1 shows the ANCOVA test statistics for the observed data and Supplementary Table S5 shows the pooled statistics from analyses of the multiple imputation datasets. Note that for perceived stress, although the CIs overlapped zero for both course types when considered separately, the intercept was significant, indicating reduction in ratings when looking across the entire sample. As for the course type differences, the effect exceeded the non-inferiority criterion *ƞ_p_^2^* < 0.01 for dispositional mindfulness; moreover, it was significant to the advantage of ReST. Course type had negligible effects on cognitive lapses and perceived stress (*ƞ_p_^2^’s* < 0.01, n.s.). The results support H2 (a–c) that the average long-term benefits of ReST were not inferior to those of CMT for any of the outcomes.

**Table 1:** ANCOVA test results for the change scores representing the difference from before to 6 months after the restoration skills training (ReST) and conventional mindfulness training (CMT) courses in ratings of dispositional mindfulness with the Five Facet Mindfulness Questionnaire (FFMQ), cognitive lapses with the Cognitive Failures Questionnaire (CFQ), and perceived stress with the Perceived Stress Scale (PSS).

		ANCOVA test results			
		*F*	*df*	*p*	*ƞ_p_^2^*
Dispositional mindfulness (FFMQ)	Corrected Model	12.75	2	<0.001	0.282
	Intercept	28.70	1	<0.001	0.306
	Pretest FFMQ score	22.97	1	<0.001	0.261
	Course type (ReST, CMT)	4.18	1	0.045	0.060
Cognitive lapses (CFQ)	Corrected Model	10.09	2	<0.001	0.237
	Intercept	11.27	1	0.001	0.148
	Pretest CFQ score	20.18	1	<0.001	0.237
	Course type (ReST, CMT)	0.31	1	0.578	0.005
Perceived stress (PSS)	Corrected Model	8.10	2	0.001	0.199
	Intercept	11.30	1	0.001	0.148
	Pretest PSS score	16.19	1	<0.001	0.199
	Course type (ReST, CMT)	0.01	1	0.941	0.000

ReST *n* = 37, CMT *n* = 31. Degrees of freedom for the error terms = 65.

### H3: Proportions of Participants Showing Reliable Improvements or Deterioration

According to the overall reliable change classifications building on the reliable change index applied to the observed data, 35% of the ReST participants and 39% of the CMT participants demonstrated reliable improvement. The likelihood of reliable improvement was similar in ReST and CMT, with *χ*^2^(1, *N* = 68) = 0.93, *p* = 0.761, *φ* = 0.037. Analyses of the multiple imputation datasets corroborated the conclusion [ReST_pooled_ = 36%, CMT_pooled_ = 40%; *χ*^2^_pooled_ (1, *N* = 97)=0.18, *p* = 0.676, mean *φ* = 0.037]. The results support the non-inferiority of ReST to CMT regarding the proportions of participants showing reliable improvement (H3a) 6 months after the course.

However, 11% of the responding ReST participants and 16% of the responding CMT participants showed deteriorated psychological functioning. The likelihood of reliable deterioration was, again, similar in ReST and CMT, with *χ*^2^(1, *N* = 68) = 0.42, *p* = 0.519, *φ* = 0.078. Again, analyses of the multiple imputation datasets corroborated the conclusion [ReST_pooled_ = 13%, CMT_pooled_ = 17%; *χ*^2^_pooled_ (1, *N* = 97)=0.36, *p* = 0.550, mean *φ* = 0.061]. The results thus support the non-inferiority of ReST to CMT regarding the proportions of participants showing reliable deterioration (H3b) 6 months after the course.

### H4: Proportions of Participants Who Continued to Practice Mindfulness

Table 2 shows distributions and full test statistics for the forms of practices and frequencies with which follow-up responders reported having continued to practice, and Supplementary Table S6 gives the same information for the analyses of the multiple imputation datasets. It can be seen that continued formal meditation was relatively uncommon while larger numbers continued with informal meditation and/or kept using mindfulness in daily life situations. The analyses tested hypotheses regarding superiority (rather than non-inferiority) of ReST over CMT in the rates of continued practice.

**Table 2:** Observed and expected numbers and percentages of participants in restoration skills training (ReST) and conventional mindfulness training (CMT) courses who 6 months after the course indicated that they had continued to practice mindfulness occasionally (i.e., at least several times) since the course ended and regularly (i.e., at least once per week).

		Occasional practice		Regular practice	
		ReST	CMT	ReST	CMT
Any form of practice	Obs (Exp)	34 (29.8)	20 (24.2)	13 (13.8)	12 (11.2)
	% of the sample	92%	67%	35%	40%
	Test statistics	*(χ*^2^ = 6.74, *p* = 0.009, *φ* = 0.317)		*(χ*^2^ = 0.17, *p* = 0.682, *φ* = 0.050)	
Formal practice	Obs (Exp)	15 (13.8)	10 (11.2)	1 (1.7)	2 (1.3)
	% of the sample	41%	33%	3%	7%
	Test statistics	*(χ*^2^ = 0.37, *p* = 0.544, *φ* = 0.074)		*(χ*^2^ = 0.61, *p* = 0.583^*^, *φ* = 0.095)	
Informal practice	Obs (Exp)	29 (25.4)	17 (20.6)	8 (7.2)	5 (5.8)
	% of the sample	78%	57%	22%	17%
	Test statistics	*(χ*^2^ = 3.63, *p* = 0.057, *φ* = 0.233)		*(χ*^2^ = 0.26, *p* = 0.610, *φ* = 0.062)	
Daily life	Obs (Exp)	32 (28.2)	19 (22.8)	9 (10.5)	10 (8.5)
	% of the sample	87%	63%	24%	33%
	Test statistics	*(χ*^2^ = 4.89, *p* = 0.027, *φ* = 0.270)		*(χ*^2^ = 0.66, *p* = 0.416, *φ* = 0.099)	

*Fisher’s Exact *p*. Data are given for the composite classification including all three forms of practice, and separately for formal practice, informal practice, and use of mindfulness in daily life. Note that the classification of occasional practice includes all participants who reported having practiced “several times” or more, including those who reported practicing regularly. Chi-square test statistics are given for each group contrast. All analyses comprise 37 ReST participants and 30 CMT participants (1 CMT participant was excluded due to missing practice data), although observed and expected numbers are only shown for those participants who endorsed having practiced in the given form and frequency. Each analysis has one degree of freedom.

In support of H4a, 92% of ReST participants reported having continued to practice mindfulness in some form at least occasionally (versus discontinuing the practice) after the course compared to 67% of CMT participants: a moderate-sized, significant difference, with *χ*^2^(1, *N* = 67) = 6.74, *p* = 0.009, *φ* = 0.317. Analyses of the multiple imputation datasets yielded a slightly smaller average effect size but corroborated the conclusion [ReST_pooled_ = 91%, CMT_pooled_ = 72%; *χ*^2^_pooled_ (1, *N* = 97) = 4.71, *p* = 0.030, mean *φ* = 0.241]. Among the specific types of continued occasional practice, use of mindfulness in daily life was significantly more common among ReST participants (see Table 2).

The participants who reported some form of continued mindfulness practice on a regular basis comprised 35% for ReST and 40% for CMT, with *χ*^2^(1, *N* = 67) = 0.17, *p* = 0.682, *φ* = 0.050, which does not support H4b regarding superiority of ReST over CMT in continued regular practice. Analyses of the multiple imputation datasets yielded the same conclusion [ReST_pooled_ = 35%, CMT_pooled_ = 42%; *χ*^2^_pooled_ (1, *N* = 97) =0.42, *p* = 0.528, mean *φ* = 0.069]. Similarly, the further analyses of the different types of continued regular practice did not show any significant advantage for ReST over CMT.

### The Role of Continued Practice in Sustaining Improvements Achieved With the Course

In the following, we describe the relationships between improvement achieved with the course, continued occasional and regular practice after the course, and improvement observed 6 months after the course. We do not consider deterioration either with the course or at follow-up because the number of deteriorated participants was too small for such contrasts.

#### Predicting Continued Use of Mindfulness

Improvements observed directly after the course interacted with Course type to predict continued use of mindfulness in the following 6 months (see Table 3). Looking first at how many continued to practice at least occasionally (versus discontinuing the practice), we saw that of those participants who had not demonstrated improvement directly after the course, 85% of the ReST participants and 71% of the CMT participants reported such practice (for the imputed datasets: ReST_pooled_ = 85%, CMT_pooled_ = 75%). ReST and CMT participants who had not demonstrated improvement with the course were thus relatively highly and similarly likely to continue with at least occasional practice. In contrast, the pattern differed with a large effect size for those participants who had demonstrated improvement directly after the course: of these, 100% of the ReST participants but only 58% of the CMT participants continued to practice at least occasionally (for the imputed datasets: ReST_pooled_ = 100%, CMT_pooled_ = 68%). Improvement with the course was positively related to continued practice for ReST participants but not for CMT participants.

**Table 3:** The relationship between improvement in psychological functioning observed directly after the course, as determined by a reliable change index, and continued mindfulness practice in the following 6 months.

		Continued to practice after course									
		At least occasionally					Regularly				
Improved with course		ReST	CMT	*χ^2^*	*p*	*φ*	ReST	CMT	*χ^2^*	*p*	*φ*
Obs.	No	17 (exp. 15.7), *n* = 20	12 (exp. 13.3), *n* = 17	1.13	0.428^*^	0.174	2 (exp. 5.9), *n* = 20	9 (exp. 5.1), *n* = 17	8.11	0.004	0.468
	Yes	14 (exp. 11.3), *n* = 14	7 (exp. 9.7), *n* = 12	7.22	0.012^*^	0.527	10 (exp. 6.5), *n* = 14	2 (exp. 5.5), *n* = 12	7.80	0.005	0.548
MI	No	26.8, *n* = 31.7	19.3, *n* = 25.7	0.70	0.402	0.119	5.0, *n* = 31.7	13.3, *n* = 25.7	6.40	0.012	0.387
	Yes	21.2, *n* = 21.3	12.4, *n* = 18.3	7.15	0.008	0.441	13.5, *n* = 21.3	5.0, *n* = 18.3	3.96	0.047	0.363

* Fisher’s Exact p.

Observed and expected values and chi square test statistics are given separately for at least occasional practice (i.e., having practiced “Several times” or more versus “Never” or “Just the odd occasion”) and regular practice (i.e., at least once per week), as well as for analyses of the observed (Obs.) data and of the 30 multiple imputation (MI) datasets. For analyses of the observed data, *N* = 63 because five participants lacked data on either improvement directly after the course or continued practice. For analyses of the multiple imputation datasets, *N* = 97.

Looking at continued regular practice, we saw that of those participants who had not demonstrated improvement directly after the course, only 10% of the ReST participants but 53% of the CMT participants continued to practice mindfulness regularly (for the imputed datasets: ReST_pooled_ = 16%, CMT_pooled_ = 52%). CMT participants were thus more likely to continue to practice even though they had not achieved any apparent benefit from the course. In stark contrast, for participants who had demonstrated improvement directly after the course, 71% of ReST participants compared to only 17% of CMT participants continued to practice regularly. The difference was somewhat less pronounced but still significant in analyses of the imputed datasets (ReST_pooled_ = 63%, CMT_pooled_ = 27%). These moderate to large group differences were thus in opposite directions depending on whether improvement had been achieved with the course: improvement with the course was strongly positively related to continued regular practice among ReST participants while CMT participants were, unexpectedly, particularly unlikely to continue with regular practice if they had demonstrated improvement directly after the course.

#### Predicting Improvement at Follow-Up

Improvement observed directly after the course was differently related to improvement at follow-up depending on Course type. Among ReST participants, 71% (ReST_pooled_ = 66%) of those who had demonstrated improvement directly after the course continued to show improvement at follow-up: a strong and significant relationship [*χ*^2^(1, *N* = 34) = 11.10, Fisher’s Exact *p* = 0.001, *φ* = 0.571; for the imputed datasets: *χ*^2^_pooled_ = 9.75, *p* = 0.002, mean *φ* = 0.506]. For CMT, however, that proportion was 58% (CMT_pooled_ = 54%): improvement directly after the course was not significantly related to improvement at follow-up [though the effect was of moderate size: *χ*^2^(1, *N* = 30) = 2.80, Fisher’s Exact *p* = 0.136, *φ* = 0.306; for the imputed datasets: *χ*^2^_pooled_ = 2.09, *p* = 0.149, mean *φ* = 0.246]. ReST participants were thus likely to sustain improvements achieved during the course over the 6 months after the course, whereas CMT participants’ psychological functioning at follow-up was not clearly related to any improvements achieved in the course.

Continued use of mindfulness was also differently related to improvement at follow-up depending on Course type, but only for continued regular practice. Looking first at continued at least occasional practice (versus discontinuing the practice), such practice was so common among ReST participants that it could not meaningfully differ between improved and non-improved parts of the sample: 100% (pooled estimate = 98%) of those ReST participants who demonstrated improvement at follow-up had continued to practice at least occasionally, but so had 88% (pooled estimate = 86%) of those who did not demonstrate improvement at follow-up [*χ*^2^(1, *N* = 37) = 1.77, Fisher’s Exact *p* = 0.297, *φ* = 0.219; for the imputed datasets: *χ*^2^_pooled_ = 1.70, *p* = 0.193, mean *φ* = 0.202]. For CMT, those proportions were somewhat lower: 75% of those who demonstrated improvement at follow-up had practiced at least occasionally vs. 61% of those who did not demonstrate improvement [*χ*^2^(1, *N* = 30) = 0.63, Fisher’s Exact *p* = 0.694, *φ* = 0.144; for the imputed datasets: *χ*^2^_pooled_ = 0.46, *p* = 0.496, mean *φ* = 0.114]. Continued at least occasional practice was thus not clearly related to improvement at follow-up.

Looking at continued regular practice, however, we saw that among ReST participants, 62% (ReST_pooled_ = 57%) of those who demonstrated improvement at follow-up had continued to practice regularly *χ*^2^(1, *N* = 37) = 6.13, Fisher’s Exact *p* = 0.028, *φ* = 0.407; for the imputed datasets: *χ*^2^_pooled_ = 4.21, *p* = 0.041, mean *φ* = 0.321. For CMT participants, that proportion was 42% [CMT_pooled_ = 43%; *χ*^2^(1, *N* = 30) = 0.02, Fisher’s Exact *p* = 1.00, *φ* = 0.028; for the imputed datasets: *χ*^2^_pooled_ = 0.26, *p* = 0.613, mean *φ* = 0.052]. Continued regular practice was thus moderately related to improvement observed at follow-up for ReST participants but had a weak and non-significant relationship with improvement at follow-up for CMT participants.

## Discussion

Six months after completing the ReST course, former participants on average still reported greater dispositional mindfulness and fewer cognitive lapses than before the course. In contrast to the ReST participants, CMT participants’ reports did not reflect improved dispositional mindfulness on average, although they did report fewer cognitive lapses. The effects on perceived stress, that were already less consistent directly after the course (Lymeus et al., 2020) were not sustained for either course type.

Looking at change in psychological functioning at the individual level, the likelihoods of experiencing reliable improvement or deterioration in one or more of the assessed domains of psychological functioning 6 months after the course were similar in ReST and CMT. The proportion of follow-up responders that showed improvement after 6 months (35 and 39% for ReST and CMT, respectively) was quite similar to the proportion Lymeus et al. (2020) observed directly after the course (38% of the course completers). In other words, ReST was at least as effective as the conventional approach to mindfulness training for these outcomes.

The proportion that showed deteriorated functioning at follow-up (13%) was somewhat larger than that observed directly after completing the course (7%; Lymeus et al., 2020), as could be expected given that the passage of time likely introduced additional sources of variation in terms of rates of continued practice as well as extraneous factors. However, the likelihood of deterioration was not apparently larger than observations reported in other mindfulness studies or that could be expected in passive control conditions (see Baer et al., 2019). Despite using stringent criteria, we could not see any disadvantage for ReST compared to CMT in the average degree or likelihood of showing improved psychological functioning, nor in the likelihood of deterioration. These results were all corroborated in separate analyses of multiple imputation data that accounted for those participants who did not respond to the follow-up, as were the following results regarding continued practice.

ReST participants were more likely than CMT participants to keep practicing mindfulness at least occasionally (versus discontinuing the practice). This difference in long-term compliance was similar in magnitude to the moderate-sized advantages in course completion and homework practice consistency that Lymeus et al. (2020) observed during the course. Looking at specific types of continued practice, ReST participants were more likely to still be using the mindfulness skills they learned in the course in daily life situations. CMT participants were more likely to have abandoned their practice. However, the smaller subsets of participants who continued to practice regularly (i.e., at least once per week) after the course were comparable between ReST and CMT. Few in either group continued with regular formal meditation while continued informal meditation and use of mindfulness in daily life were more common.

In particular, those ReST participants who had demonstrated improved psychological functioning directly after completing the course were highly likely to keep practicing after the course, 100% of them reporting at least occasional continued practice and 71% of them reporting continued regular practice. In contrast, CMT participants showed an unexpected negative association between improvement with the course and continued practice: of those who had demonstrated improvement directly after the course, little more than half (58%) continued to practice even occasionally and only 17% continued to practice regularly. Given the relatively small *N* in this contrast, the exact proportions should be taken as tentative, but the effect was significant and corroborated in the larger multiple imputation datasets. Apparently, having achieved improved psychological functioning with the course motivated CMT participants to reduce or discontinue their practice rather than sustain it. This aligns with the understanding that conventional mindfulness practice requires effort: once improvements were achieved, making further investments in mindfulness practice was less attractive. Intriguingly, CMT participants who had not improved with the course were much more likely to continue to practice. This could possibly reflect a ceiling effect where those CMT participants who were relatively capable already before the course had limited room for further improvement but high ability and motivation to practice. In contrast, having achieved improvements through ReST training apparently motivated participants to continue practicing. This aligns with the understanding that ReST practices promote restoration and hence can be more immediately rewarding to use.

Restoration skills training participants were also likely to sustain the improvements they had achieved in the course over the following 6 months whereas CMT participants’ psychological functioning at follow-up was not as clearly related to improvements achieved in the course. ReST participants also showed a relationship between continued regular practice and improvement demonstrated at follow-up, whereas CMT participants’ likelihood of demonstrating improvement at follow-up was unrelated to whether they had continued to practice mindfulness. Continued at least occasional practice was very common among ReST participants, both among those who demonstrated improvement at follow-up and among those who did not.

The continued use of mindfulness following ReST could be due to the previously reported restorative short-term effects, the greater ease of ReST practices compared with CMT (Lymeus et al., 2018), and the more stable commitment to meditation training seen during the course (Lymeus et al., 2019). Noting that it was the rate of “use in daily life” rather than of formal or informal practice that was significantly higher among ReST participants, however, it is also possible that the connection of the practices to natural settings contributed by promoting learned associations between nature and mindfulness. Assuming the operation of context dependent learning (*cf.* Smith and Vela, 2001) and conditioning processes in restorative nature experience (see Egner et al., 2020), one could expect that having learned mindfulness in nature would make mindfulness experiences and practices more readily available in naturalistic day-to-day nature contacts. Referring to natural settings, Kaplan (2001) similarly theorized that people can learn from experience to notice and connect with setting characteristics that support meditative states and practices. The idea that people learn to turn to different environments for needed psychological support is also represented in Korpela’s work on environmental self-regulation (e.g., Korpela et al., 1992, 2018), where natural settings are emphasized together with some built environments of specific personal relevance. Conventional mindfulness training completed in commonplace indoor settings—where many people spend most of their time and often experience various demands and stressors, where environmental support for restoration and mindfulness is typically weak, and where the practice approach is largely disconnected from the setting—would not likely produce such specific learned connections between the practice and given setting characteristics.

### Strengths and Limitations

Few mindfulness studies and very few studies on nature-based health interventions have followed participants’ long-term commitment and development after treatment. This study follows up on our randomized contrast between two active and formally matched interventions (ReST, CMT). It also connects relevant mechanisms (i.e., continued practice) with improvements in broad domains of psychological functioning. While the follow-up addresses a distinct set of hypotheses and adds to the larger project of which it is part, it necessarily builds on participants, methods, and findings from previous studies.

Given the lower drop-out from ReST than from CMT (Lymeus et al., 2019), we focused the follow-up analyses on course completers to gain an understanding of the expected benefits after completing the respective courses: Although the baseline values of follow-up responders and the other participants were fairly balanced, we used baseline values together with other relevant variables in the imputation model and followed the recommended practice of using ANCOVA with baseline values as a covariate to analyze average outcomes (e.g., Vickers and Altman, 2001). Our analyses of the multiple imputation datasets corroborated the findings in the observed data. Yet, all analyses were done per-protocol, including only course completers. As in applied contexts, completion of the course was voluntary and thus potentially influenced by the course experience to some extent. Furthermore, information about the different course conditions could possibly have leaked between participants who were all students at the same university and some of whom were likely acquainted, although we asked them not to discuss the course or study outside of their course group. However, we consider that the demand characteristics should be similar in the two courses because all participants received *bona fide* mindfulness training and they were not informed about the specific aims of the study. Furthermore, assuming that the more decimated sample that completed CMT, and eventually responded to the follow-up, could have been more motivated or capable of mindfulness training than those who dropped out, as suggested by Lymeus et al. (2019), comparisons between only the course completers should be to the advantage of CMT over ReST.

Follow-up of the course dropouts would have provided additional value had it been possible (Nam and Toneatto, 2016; Sekhon et al., 2017; Baer et al., 2019). Furthermore, including a passive control condition (as was done in the evaluation of outcomes directly after the course, but only in the fourth data collection round; see Lymeus et al., 2020) would have allowed us to support conclusions regarding improved levels of psychological functioning with between-subjects contrasts in addition to the within-subjects contrasts we rely on here. However, we were not able to retain and motivate the passive control participants from data collection round 4 to also respond to the follow-up 6 months later. Complementing analyses of average effects with analyses of the reliable change index classifications, which affirm with 95% confidence that actual change has occurred, bolster the validity of the within-subjects contrasts.

We evaluated the non-inferiority of ReST compared to a conventional course in achieving long-term benefits for psychological functioning, which is the appropriate way of assessing the utility of a modified treatment (e.g., in terms of a more efficient or acceptable delivery) relative to an established treatment (Snapinn, 2000; Schumi and Wittes, 2011). Even with the evidence of particular advantages connected to the setting and delivery of ReST compared with CMT (i.e., higher perceived restorativeness and state mindfulness, higher course compliance and more sustained practice habits, as reported in Lymeus et al., 2019), using the more familiar superiority approach to contrast two formally matched, *bona fide* mindfulness training courses would have been unlikely to produce substantive and reliable differences in distal assessments of broad domains of psychological functioning (although we did find one that we had not hypothesized, for dispositional mindfulness). The non-inferiority approach allowed us to conclude that ReST had no meaningful disadvantages in terms of sustained benefits for psychological functioning. Hence, the modifications that enhanced the acceptability of ReST did not compromise its effectiveness compared with the more demanding conventional approach. We can therefore draw the non-trivial conclusion that the advantages achieved with the ReST approach are worthwhile.

We conducted several statistical tests, some with quite low power. The tests for H1 expected average benefits for both ReST and CMT and thus did not contribute to any inflated risk of spurious effects to the advantage of ReST. In the tests for non-inferiority (H2–H3), the inferences drawn do not rest on statistical significance but on the absolute magnitude of the observed effect size. Hence, any increase in the risk of spurious effects was strictly to the disadvantage of ReST (see Snapinn, 2000; Schumi and Wittes, 2011). The two tests for significant differences in sustained mindfulness practice (H4) relied on explicit hypotheses. Although the tests to reveal in what form(s) of continued practice ReST and CMT participants differed and the tests to explain relations between continued practice and sustained improvement largely aligned with relevant theory and previous findings, they should be taken as exploratory.

While the three measures of psychological functioning are well-established and valid (Cohen, 1988b; Sauer et al., 2013; Carrigan and Barkus, 2016), each has been debated (Grossman and Van Dam, 2011; Nielsen et al., 2016; Könen and Karbach, 2018). Assessments of behavioral, performance-based, and psychophysiological operationalizations of the different domains of psychological functioning could have provided additional insights. As for our measures of continued practice, they were designed to be quick and easy to answer reliably in retrospect, but we cannot verify their correspondence with actual behavior. Also note that we, building on Lymeus et al. (2020), evaluated non-inferiority based on observed effect sizes rather than the confidence intervals around observed effects, which would have required a much larger sample. Future work with improved assessment methods and larger samples would help progress ReST research.

The ReST classes in this study were held in an urban botanical garden, which served as a convenient example of a natural setting with high restorative quality, although it is to some extent disturbed by traffic noise and visual contact with surrounding urban structures. The Uppsala botanical garden is freely available to the student population, and those who study in nearby campuses can visit it to meditate or, as more commonly observed, simply relax during study breaks; hence, using the botanical garden contributes to the ecological validity and practical relevance of this study. However, using this relatively familiar setting, with its particular qualities, may also have contributed to shaping the practice experience and outcomes in certain ways. It remains for future studies to investigate how the ReST approach works in other settings. We also see several other future research directions for ReST.

### Future Directions

We consider that the ReST approach should be applicable in a range of different settings that are rich in natural features (*cf.* Macaulay et al., 2022). In fact, the ReST approach is intended to be robust to different environmental constraints (e.g., the mechanical noise from the climate system in the greenhouse) in similar ways that ReST and other mindfulness approaches guide participants to accept thought intrusions and other personal discomforts; however, it has yet to be tested if it can reduce sensitivity to ambient stressors in urban nature settings. To extend the possible applications of ReST, studying practice in other natural settings (e.g., more constrained, wilder, virtual) could also be considered. So could remote delivery *via* mobile technologies.

As for target populations, we have shown that ReST is promising for university students with stress and concentration problems. Extensions could target other people who could benefit from an undemanding way to lastingly bolster psychological functioning, including children, specific diagnostic groups, and care-giving professionals. Furthermore, investigating potential benefits of ReST training beyond individual health could extend its scope of use, for instance to foster positive and sustainable human-nature relations (*cf.* Geiger et al., 2019; Lymeus et al., 2022; Macaulay et al., 2022) or interpersonal relations (see Hartig, 2021). We consider that such potential benefits may evolve over even longer time-spans than benefits related to individual health.

Studies comparing ReST to other activities in natural settings (e.g., regular visits without any mindfulness-based instructions) could isolate the relative contributions of the setting and the practice to its effects. Studying the qualitative aspects of the ReST experience and the progression of relevant processes in ReST meditations and courses would further develop the understanding of what differentiates ReST from CMT and from nature contacts without meditation practice.

### Conclusion

The findings help fill multiple gaps in meditation research (Davidson and Kaszniak, 2015; Van Dam et al., 2018): few intervention studies have compared active meditation conditions, reported long-term follow-up data, or investigated factors that help explain long-term commitment and continuing benefits following an introductory mindfulness course. The findings also help advance research at the intersection of environmental psychology and allied health sciences. They indicate that careful integration of environmental and individual-level approaches can not only achieve added short-term benefits, such as cognitive and emotional restoration, over a comparable intervention without environmental support, but may also help sustain changes in relevant habits, skills, and general psychological functioning.

Finally, the findings add to previous reports regarding ReST (Lymeus et al., 2018, 2019, 2020) and provide further support for the utility of ReST as a potential health intervention. Even without any particular instructions or reminders to keep practicing, many ReST participants apparently found the skills taught during the course useful and appealing enough to keep practicing and applying them in their daily lives. Many of the participants were also able to sustain the improvements they had achieved in the course over the following 6 months. This is important because any personal or societal investment in a health intervention must be motivated by confidence in its ability to achieve sustainable benefits. This study is an important step toward building such confidence in ReST.

## Data Availability Statement

The datasets presented in this study will be made available following a 2-year embargo period, through the Swedish National Data Service (https://doi.org/10.5878/prw6-k648). Further inquiries can be directed to FL (freddie.lymeus@ibf.uu.se).

## Ethics Statement

This study involving human participants was reviewed and approved by the Regional Ethical Review Board for Uppsala, ref. no: 2013/033. The participants provided their written informed consent to participate in this study.

## Author Contributions

FL designed the study and led the data collection with guidance from PL and TH. FL analyzed the data and drafted the manuscript. MW, PL, and TH contributed to the article with critical revisions. All authors approved the submitted version.

## Funding

This work was instrumentally supported by the Institute for Housing and Urban Research and the Department of Psychology, Uppsala University.

## Conflict of Interest

The authors declare that the research was conducted in the absence of any commercial or financial relationships that could be construed as a potential conflict of interest.

## Publisher’s Note

All claims expressed in this article are solely those of the authors and do not necessarily represent those of their affiliated organizations, or those of the publisher, the editors and the reviewers. Any product that may be evaluated in this article, or claim that may be made by its manufacturer, is not guaranteed or endorsed by the publisher.

## Footnotes

^1^ https://doi.org/10.5878/prw6-k648
