# Supplementary material for: Restoration Skills Training in a Natural Setting Compared to Conventional Mindfulness Training: Sustained Advantages at a 6-Month Follow-Up
Source: Front Psychol. 2022 Aug 1;13:763650. doi: 10.3389/fpsyg.2022.763650 (PMC9376351; doi:10.3389/fpsyg.2022.763650)
Supplement: Supplementary file 1 [file Data_Sheet_1.docx]

| *Table S1. Average scores and standard deviations observed before the start of the restoration skills training (ReST) and conventional mindfulness training (CMT) courses, for 139 participants: 68 who eventually participated in the 6-month follow-up survey and 71 who did not. The total sample includes all participants who started either course in any of the data collection rounds of the original study, even the 42 who were not contacted for the follow-up because they had failed to complete the course or because we did not have resources in place to conduct follow-up assessments with participants in data collection round 1 (of 4).* | | | | | |
| --- | --- | --- | --- | --- | --- |
|  |  | Course | *N* | *M* | *SD* |
| **Five Facet Mindfulness**  **Questionnaire** | **Did not participate in follow-up** | CMT | 40 | 3.04 | 0.47 |
|  |  | ReST | 31 | 3.04 | 0.44 |
|  | **Participated in follow-up** | CMT | 31 | 2.95 | 0.48 |
|  |  | ReST | 37 | 3.03 | 0.36 |
|  |  | **Total** | **139** | **3.02** | **0.43** |
| **Cognitive Failures**  **Questionnaire** | **Did not participate in follow-up** | CMT | 40 | 1.88 | 0.48 |
|  |  | ReST | 31 | 1.74 | 0.57 |
|  | **Participated in follow-up** | CMT | 31 | 1.77 | 0.47 |
|  |  | ReST | 37 | 1.89 | 0.44 |
|  |  | **Total** | **139** | **1.83** | **0.49** |
| **Perceived**  **Stress Scale** | **Did not participate in follow-up** | CMT | 40 | 1.92 | 0.45 |
|  |  | ReST | 31 | 1.97 | 0.48 |
|  | **Participated in follow-up** | CMT | 31 | 1.97 | 0.44 |
|  |  | ReST | 37 | 1.97 | 0.56 |
|  |  | **Total** | **139** | **1.96** | **0.48** |

| *Table S2. Statistical checks on baseline balance in ratings with the Five Facet Mindfulness Questionnaire, Cognitive Failures Questionnaire, and Perceived Stress Scale before the course started. The total sample (N = 139) includes all participants who started either course in any of the data collection rounds of the original study and the follow-up sample includes those participants from data collection rounds 2 – 4 who responded to the follow-up survey. The multivariate ANOVA thus contrasts participants who following random assignment started restoration skills training (ReST; N = 68) with those who started conventional mindfulness training (CMT; N = 71) and the ReST and CMT participants who eventually responded to the 6-month follow-up (N = 68) with those for whom we did not obtain follow-up data for any reason (i.e., dropped out, not contacted, not reachable, or declined; N = 71).* | | | | |
| --- | --- | --- | --- | --- |
|  |  | ***Multivariate ANOVA test statistics*** | | |
| **Effect** | ***--*** | ***F* (3, 133)** | ***p*** | ***ƞ_p_^2^*** |
| Intercept |  | 7634.86 | .000 | .994 |
| Course type (ReST, CMT) |  | 0.29 | .835 | .006 |
| Follow-up completed (yes, no) |  | 0.29 | .833 | .006 |
| Course type * Follow-up completed |  | 1.63 | .184 | .036 |

| *Table S3. Average scores and standard deviations observed before the start of the restoration skills training (ReST) and conventional mindfulness training (CMT) courses, for 97 participants: 68 who eventually participated in the 6-month follow-up survey and 29 who were contacted for the follow-up but were unreachable or declined to participate. The total sample includes all participants who completed either course in data collection rounds 2 – 4 of the original study and thus were eligible for follow-up. Round 1 participants were not contacted for the follow-up and are omitted from these analyses (see Tables S1-2).* | | | | | |
| --- | --- | --- | --- | --- | --- |
|  |  | Course | *N* | *M* | *SD* |
| **Five Facet Mindfulness**  **Questionnaire** | **Did not participate in follow-up** | CMT | 13 | 3.06 | 0.56 |
|  |  | ReST | 16 | 2.93 | 0.43 |
|  | **Participated in follow-up** | CMT | 31 | 2.95 | 0.48 |
|  |  | ReST | 37 | 3.03 | 0.36 |
|  |  | **Total** | 97 | 2.99 | 0.43 |
| **Cognitive Failures**  **Questionnaire** | **Did not participate in follow-up** | CMT | 13 | 1.81 | 0.56 |
|  |  | ReST | 16 | 1.80 | 0.65 |
|  | **Participated in follow-up** | CMT | 31 | 1.77 | 0.47 |
|  |  | ReST | 37 | 1.89 | 0.44 |
|  |  | **Total** | 97 | 1.83 | 0.50 |
| **Perceived**  **Stress Scale** | **Did not participate in follow-up** | CMT | 13 | 1.91 | 0.50 |
|  |  | ReST | 16 | 2.10 | 0.47 |
|  | **Participated in follow-up** | CMT | 31 | 1.97 | 0.44 |
|  |  | ReST | 37 | 1.97 | 0.56 |
|  |  | **Total** | 97 | 1.99 | 0.50 |

| *Table S4. Statistical checks on baseline balance in ratings with the Five Facet Mindfulness Questionnaire, Cognitive Failures Questionnaire, and Perceived Stress Scale before the course started. The total sample (N = 97) includes all participants who completed either course in data collection rounds 2 – 4 of the original study and thus were eligible for follow-up. The multivariate ANOVA thus contrasts participants who following random assignment started restoration skills training (ReST; n = 53) with those who started conventional mindfulness training (CMT; n = 44) and the ReST and CMT participants who eventually responded to the 6-month follow-up (n = 68) with those who were contacted about the follow-up but were unreachable or declined to participate (n = 29).* | | | | |
| --- | --- | --- | --- | --- |
|  |  | ***Multivariate ANOVA test statistics*** | | |
| **Effect** | ***--*** | ***F* (3, 91)** | ***p*** | ***ƞ_p_^2^*** |
| Intercept |  | 4177.79 | .000 | .993 |
| Course type (ReST, CMT) |  | 0.29 | .834 | .009 |
| Follow-up completed (yes, no) |  | 0.06 | .983 | .002 |
| Course type * Follow-up completed |  | 0.77 | .512 | .025 |

*Table S5. Pooled test results from ANCOVA on 30 multiple imputation data sets, for the change scores representing the difference from before to six months after the restoration skills training (ReST) and conventional mindfulness training (CMT) courses in ratings of dispositional mindfulness with the Five Facet Mindfulness Questionnaire (FFMQ), cognitive lapses with the Cognitive Failures Questionnaire (CFQ), and perceived stress with the Perceived Stress Scale (PSS).*

|  |  |  | **Pooled ANCOVA test results** | | | | | | |
| --- | --- | --- | --- | --- | --- | --- | --- | --- | --- |
|  |  |  | ***F*** |  | ***df*** |  | ***p*** |  | ***ƞ_p_^2^*** |
| **Dispositional mindfulness (FFMQ)** | Corrected Model |  | 9.34 |  | 2 |  | <.001 |  | .261 |
|  | Intercept |  | 18.93 |  | 1 |  | <.001 |  | .275 |
|  | Pretest |  | 15.39 |  | 1 |  | <.001 |  | .232 |
|  | Course type (ReST, CMT) |  | 3.97 |  | 1 |  | .047 |  | .052 |
| **Cognitive lapses (CFQ)** | Corrected Model |  | 4.75 |  | 2 |  | .011 |  | .182 |
|  | Intercept |  | 5.29 |  | 1 |  | .023 |  | .100 |
|  | Pretest |  | 9.97 |  | 1 |  | .002 |  | .179 |
|  | Course type (ReST, CMT) |  | 0.40 |  | 1 |  | .528 |  | .009 |
| **Perceived stress (PSS)** | Corrected Model |  | 4.33 |  | 2 |  | .015 |  | .147 |
|  | Intercept |  | 6.63 |  | 1 |  | .011 |  | .104 |
|  | Pretest |  | 8.81 |  | 1 |  | .003 |  | .145 |
|  | Course type (ReST, CMT) |  | 0.156 |  | 1 |  | .693 |  | .003 |

Note. All analyses comprise 53 ReST participants (16 of whom were included with imputed data) and 44 CMT participants (13 of whom were included with imputed data). Degrees of freedom for the error terms = 94.

*Table S6. Pooled percentage estimates and ranges of Chi-square test statistics obtained from analyses of 30 multiple imputation datasets, comparing the proportions of former participants in restoration skills training (ReST) and conventional mindfulness training (CMT) courses who six months after the course indicated that they had continued to practice mindfulness at least occasionally (versus discontinuing the practice) and regularly (i.e., at least once per week) since the course ended. Data are given for the composite classification including all three forms of practice, and separately for formal practice, informal practice, and use of mindfulness in daily life. Note that the classification of occasional practice includes all participants who reported having practiced “several times” or more, including those who reported practicing regularly.*

|  |  |  | **Occasional practice** |  | **Regular practice** |
| --- | --- | --- | --- | --- | --- |
| **Any form of practice** | Pooled proportion estimate |  | ReST: 91%, CMT: 72% |  | ReST: 35%, CMT: 42% |
|  | Range of test statistics |  | *χ*^2^ = 4.71, *p*: = .030, mean *φ*:= .241 |  | *χ*^2^ = 0.40, *p*: = .526, mean *φ*:= .069 |
| **Formal practice** | Pooled proportion estimate |  | ReST: 42%, CMT: 38% |  | ReST: 3%, CMT: 8% |
|  | Range of test statistics |  | *χ*^2^ = 0.24, *p*: = .624, mean *φ*:= .039 |  | *χ*^2^ = 1.04, *p*: = .307, mean *φ*:= .115 |
| **Informal practice** | Pooled proportion estimate |  | ReST: 75%, CMT: 56% |  | ReST: 21%, CMT: 17% |
|  | Range of test statistics |  | *χ*^2^ = 2.84, *p*: = .093, mean *φ*:= .201 |  | *χ*^2^ = 0.36, *p*: = .551, mean *φ*:= .058 |
| **Daily life** | Pooled proportion estimate |  | ReST: 83%, CMT: 63% |  | ReST: 23%, CMT: 34% |
|  | Pooled test statistics |  | *χ*^2^ = 3.86, *p*: = .050, mean *φ*:= .225 |  | *χ*^2^ = 1.22, *p*:= .270,, mean *φ*: = .121 |

All analyses comprise 53 ReST participants (16 of whom were included with imputed data) and 44 CMT participants (14 of whom were included with imputed data). Each analysis has 1 degree of freedom.
